# Assessment of the burden of disease for patients with peripheral artery disease undergoing revascularization in England

**DOI:** 10.1177/1358863X221096704

**Published:** 2022-06-07

**Authors:** Laura Portas, Rupert Bauersachs, Kevin Bowrin, Jean-Baptiste Briere, Alexander Cohen, Maria Huelsebeck, Schuyler Jones, Jennifer K Quint

**Affiliations:** 1 National Heart and Lung Institute, Imperial College London, London, UK; 2 Department of Vascular Medicine – Angiology, Klinikum Darmstadt, Darmstadt, Germany; 3 Bayer plc, Reading, UK; 4 Bayer AG, Berlin, Germany; 5 Department of Hematological Medicine, Guy’s & St Thomas’ NHS Foundation Trust, King’s College London, London, UK; 6 Duke Clinical Research Institute, Durham, NC, USA; 7 Duke University Medical Center, Durham, NC, USA

**Keywords:** lower-limb revascularization, peripheral artery disease (PAD), healthcare resource utilization

## Abstract

**Background:** Symptoms, severity, and acuteness of peripheral artery disease (PAD) are major determinants of severe limb symptoms, subsequent risk of cardiovascular events, and mortality. Lower-extremity revascularization (LER) is a key option to relieve symptoms and to prevent limb loss in symptomatic patients with PAD. This study aimed to quantify the burden of disease among patients with PAD-LER in England. **Methods:** A retrospective population-based study of linked primary and secondary care electronic health records, included 13,869 adult patients (aged ⩾ 18 years) with PAD-LER from 2003 to 2018. The incidence of first ever PAD-LER was estimated both overall and by type of procedure (endovascular/surgical). Health resource utilization associated with PAD-related complications and treatment patterns were assessed. **Results:** A high annual incidence of lower-limb revascularization (41.2 per 1000 person years) and a nearly double incidence of endovascular first revascularization compared with open surgery were observed. More than 70% of patients with PAD-LER had a history of hyperlipidemia and hypertension and roughly one-third were diabetic and had a history of coronary artery disease. Cardiovascular mortality accounted for one-third (34.1 per 1000 person years) of all-cause mortality. Over 93% of patients were hospitalized for any reason and the commonest reasons for hospitalization were cardiovascular diseases and PAD with about one-third hospitalized for revascularization reoccurrence. **Conclusion:** There is a significant burden of PAD-LER to the individual and society with ongoing healthcare resource utilization, treatment, and increasing mortality.

## Background

Peripheral artery disease (PAD) is common, with most recent estimates suggesting prevalence has reached pandemic proportions, with more than 236 million people with PAD worldwide.^1^ Although it is generally uncommon in individuals younger than 40 years of age, it affects one in 10 individuals aged 70 years or older and one in six individuals aged 80 years or older.^2^ It is established that the symptoms, severity, and acuteness of PAD are major determinants of subsequent risk of major adverse cardiovascular events (MACE) and major adverse limb events (MALE).^3^

Independent of symptoms, patients diagnosed with PAD are at increased risk of cardiovascular (CV) death and have a 10-year all-cause mortality risk more than double compared with those without PAD.^4^

PAD due to atherosclerosis can affect any artery perfusing the lower extremities and the coincidence of PAD with atherosclerosis in other arterial beds (such as coronary artery disease (CAD) and cerebrovascular disease) characterizes patients at very high risk of CV and limb events (myocardial infarction (MI), coronary revascularization, stroke, carotid revascularization, acute limb ischemia (ALI), peripheral artery revascularization, or major amputation) who may benefit from intensive secondary preventive therapies.^5^

Although multiple studies^6–8^ have demonstrated that the management of lower-extremity PAD carries a high burden in terms of frequent CV events and hospitalizations, little evidence exists on the burden of disease among unselected patients undergoing revascularization intervention.

Therefore, this study aims to contribute towards addressing this gap by assessing and quantifying the burden of disease among patients with PAD undergoing revascularization in England.

## Methods

### Study design and data source

Pseudonymized primary care electronic records from the Clinical Practice Research Datalink (CPRD) GOLD and Aurum primary care databases were obtained. This work is based in part on data from the CPRD obtained under license from the United Kingdom (UK) Medicines and Healthcare products Regulatory Agency (MHRA). The protocol for this research was approved by an external review committee for the research data governance group (RDG) and for the MHRA Database Research (protocol number 20_008R). Generic ethical approval for observational research using CPRD with approval from RDG was granted by an HRA Research Ethics Committee (East Midlands Derby, REC reference number 05/MRE04/87). Linked pseudonymised data were provided for this study by CPRD. Datasets were linked by National Health Service (NHS) Digital, the statutory trusted third party for linking data, using identifiable data proprietary to NHS Digital. Select practices consented to this process, with individual patients afforded the right to opt out.

In this retrospective cohort analysis, data for adults (aged ⩾ 18 years) with lower-extremity PAD were examined from 2003 to 2018. Individuals with PAD were included from CPRD using specific Read codes and from Hospital Episode Statistics (HES) using the *International Classification of Diseases, 10th Revision* (ICD-10) codes. Patients transferring out of a CPRD participating general practitioner (GP) practice or whose last collection date was within a year of diagnosis were excluded. Patients were followed-up until the occurrence of any revascularization procedure, according to the Office of Population Censuses and Surveys Classification of Surgical Operations and Procedures (OPCS) revision 4.6 codes, or a major study end point (i.e., the patient disenrolled from the practice or the practice disenrolled from CPRD, the patient died, or end of the study period). This selected cohort of patients (i.e., the incidence of disease cohort) was used to calculate incidence of first-time ever PAD-related revascularization (overall and by type of procedure). Patients entered the second cohort (i.e., the intervention for disease cohort) if they underwent a PAD-related revascularization in addition to meeting the minimum age and data quality requirements. The second cohort was used to estimate the incidence of revascularization reoccurrence, the risk/incidence of PAD-related complications, as well as to describe healthcare resource utilization (HRU) and treatment patterns. Differences in disease complications, HRU, and treatment patterns were also explored among subgroups of patients with PAD-related revascularization.

### Outcomes

The incidence of first ever PAD-related revascularization over the study period (2003–2018) was estimated overall and by type of procedure (endovascular or surgical). Patients were considered to have had a PAD-related complication if they had, on or after the index date (i.e., date of first ever revascularization), one of the following conditions (determined using Read codes or ICD-10 codes): revascularization reoccurrence, bleeding, stroke, any amputation above/below the ankle, ALI, MI, venous thromboembolism (VTE), all-cause hospitalization, CV hospitalization, CV death, and all-cause mortality.

Medication patterns including medications acting on the renin-angiotensin system, angiotensin-receptor/neprilysin inhibitors, anticoagulants, antithrombotics/antiplatelets, beta-blockers, calcium channel blockers, and lipid-modifying agents, were described during the baseline period (i.e., 1 year prior to the first revascularization) and over follow-up (on or after the first revascularization).

Healthcare resource utilization related to PAD complications was described over the study period (2003–2018) by category of HRU (i.e., primary care, outpatient specialist visits, emergency room visits, and hospitalizations).

### Statistical analyses

Patients with incident PAD-related revascularization were described according to demographics and clinical characteristics. In a time-window of 3 years before the first revascularization (index date), the closest measure to index date was considered. Socioeconomic status was reported using indices of multiple deprivation (IMD) 2015 quintiles, with quintile 1 being the least and quintile 5 the most deprived. Significant past medical history was reported for any of the following conditions: diabetes mellitus (DM), prior ischemic or hemorrhagic stroke, chronic kidney disease (CKD), chronic obstructive pulmonary disease (COPD), heart failure (HF), hypertension, bleeding disorders, CAD.

The frequency distribution (number and percentage of patients) for categorical variables and descriptive statistics (mean, SD) for continuous variables were calculated using the denominator with nonmissing values for that variable. Missing data were described by reporting the proportion of missing data for each variable.

The incidence rate was calculated separately for incident or ‘new’ cases of PAD-related revascularizations over the study period (2003–2018). The incidence of first PAD-related lower-extremity revascularization (LER) by type of procedure and revascularization reoccurrence (after the first occurrence) was also estimated.

The incidence rate and the relative risk of individual PAD complications were estimated over the study period. Incidence rates were reported per 1000 person-years (PYRs) with accompanying 95% CIs. The relative risk of complications was compared across each patient subgroup of interest using Cox regression. Adjusted models including potential confounders as demographics, medical history, and past-year medication use were built iteratively by using a forward stepwise procedure with a 5% level of significance required to add and a 1% level of significance required to remove predictors from the model. Each predictor was checked for collinearity and dropped from the model if collinearity was found.

The adjusted hazard ratios with associated 95% CIs were derived for each subgroup and the proportional hazards assumption was tested using Schoenfeld residuals.

Each type of HRU was described from the first PAD-LER onwards. Inpatient/outpatient encounters were reported as the number and proportion of patients who had one or more visits as categories (i.e., 1–4, 5–9, 10+). The first reason for hospitalizations was considered and the mean (SD) and median (IQR) length of stay were reported (over all hospitalizations over the study period of interest). For treatments, the number and proportion of patients with one or more prescriptions for each medication class were reported during the year prior to the index date and on or after the index date. Among the subgroups of interest, statistically significant differences (*p* < 0.05) for HRU and treatment patterns were assessed by chi-squared test. Data management and analyses were performed using Stata 16.

## Results

**Figure 1.**
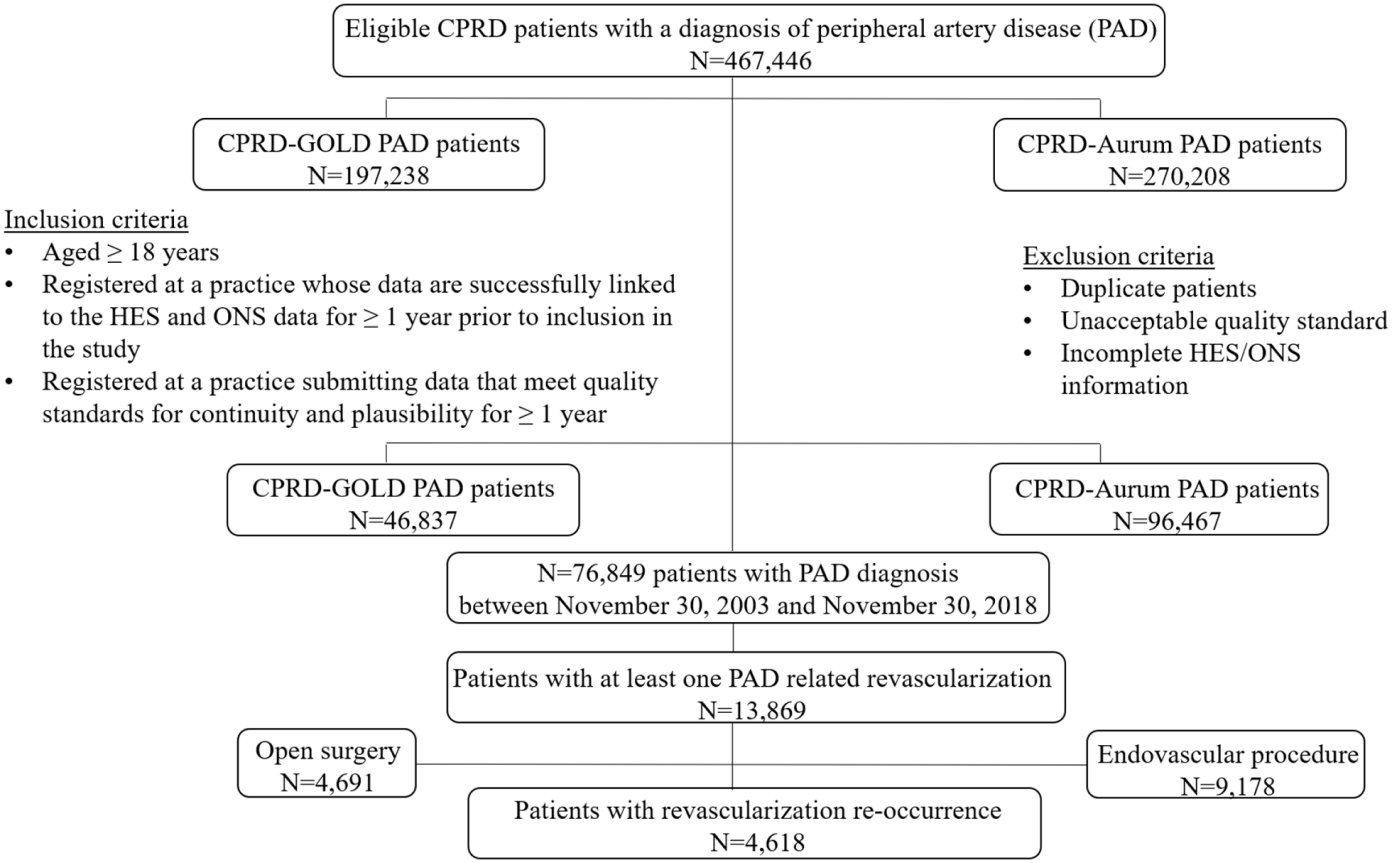
Flow chart for identification of patients with PAD and PAD-LER. CPRD, Clinical Practice Research Datalink; HES, Hospital Episode Statistics; LER, lower-extremity revascularization; ONS, Office for National Statistics.

Out of 76,849 patients with a PAD diagnosis between November 30, 2003 and November 30, 2018, 13,869 had at least one PAD-LER, with 4691 and 9178 patients who underwent an open surgery and endovascular procedure, respectively. Also, 4618 patients had repeat revascularization (Figure 1).

**Figure 2.**
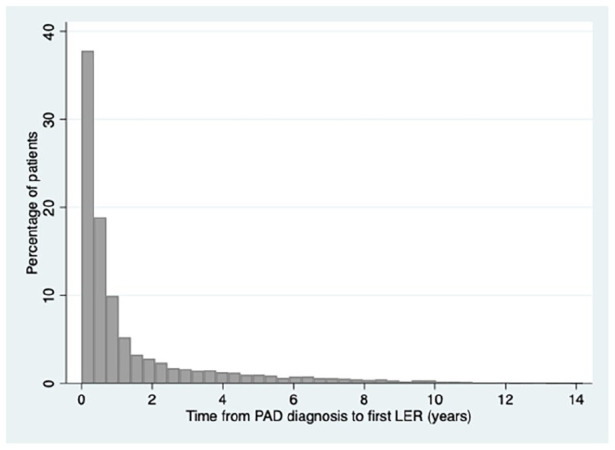
Percentage of patients who underwent LER over the years after PAD diagnosis. LER, lower-extremity revascularization; PAD, peripheral artery disease.

Time from PAD diagnosis to first LER is comprised between 1 day and 14.2 years with a mean of 1.6 (SD: 2.4) years. Half of patients had revascularization 6.6 months after PAD diagnosis with IQR 2.3 months to 1.7 years (Figure 2).

### Patient characteristics

Patients with PAD undergoing revascularization were mostly men (65.6%) and had a mean age of 69.5 years. These patients had a high mean body mass index (BMI: 27 kg/m^2^) and were mostly current smokers. Also, an increasing trend in the proportion of people with PAD-LER was observed in lower-socioeconomic groups. Hyperlipidemia and hypertension were particularly common (more than 70% of patients had a history of these conditions) and roughly one-third of patients were diabetic and had a history of CAD. Among patients with CAD, 62% had angina and 45% had MI. Looking at the characteristics of the patients by type of revascularization, the two groups confirm the same trend (i.e., age, anthropometrics, socioeconomic and smoking status) observed in the total sample. However, the group of patients who underwent an open surgery procedure is represented by a higher percentage of men (70%) compared to those with endovascular revascularization (63%). The endovascular group has a higher percentage of history of hyperlipidemia, DM, CKD, heart failure, bleeding, and CAD. Instead, patients undergoing open surgery revascularization have a higher percentage of history of stroke and, in those with CAD, of angina and MI (Table 1).

**Table 1.** Demographic and clinical characteristics of patients.

	All patients (*n* = 13,869)	Open surgery (*n* = 4691)	Endovascular (*n* = 9178)
Age, years, mean (SD)	69.5 (11.1)	69.1 (11.0)	69.7 (11.2)
**Sex, *n* (%)**			
Female	4770 (34.4)	1401 (29.9)	3369 (36.7)
Male	9099 (65.6)	3290 (70.1)	5809 (63.3)
**Socioeconomic quintile, *n* (%)**			
Quintile 1	2408 (17.4)	803 (17.1)	1605 (17.5)
Quintile 2	2553 (18.4)	877 (18.7)	1676 (18.3)
Quintile 3	2736 (19.7)	949 (20.2)	1787 (19.5)
Quintile 4	2841 (20.5)	953 (20.3)	1888 (20.6)
Quintile 5 (most deprived)	3317 (23.9)	1105 (23.6)	2212 (24.1)
Missing data	0.1%	0.1%	0.1%
**Anthropometric measures, mean (SD)**			
Weight, kg	76.9 (17.2)	76.9 (17.0)	76.9 (17.2)
Missing data	22.8%	24.0%	22.2%
Height, m	1.69 (0.1)	1.69 (0.1)	1.68 (0.1)
Missing data	25.4%	26.6%	24.7%
Body mass index, kg/m^2^	27.0 (5.3)	26.8 (5.1)	27.1 (5.4)
Missing data	25.5%	26.8%	24.8%
**Smoking, *n* (%)**			
Current smokers	5197 (37.4)	1846 (39.4)	3351 (36.5)
Ex-smokers	4336 (31.3)	1441 (30.7)	2895 (31.5)
Never smokers	4336 (31.3)	1404 (29.9)	2932 (32.0)
**Alcohol use, *n* (%)**			
Drinker	3102 (22.4)	1079 (23.0)	2023 (22.0)
Nondrinker	10,767 (77.6)	3612 (77.0)	7155 (78.0)
**Medical history, *n* (%)**			
Hyperlipidemia	10,927 (78.8)	3659 (78.0)	7268 (79.2)
Diabetes mellitus	4175 (30.1)	1300 (27.7)	2875 (31.3)
Prior ischemic or hemorrhagic stroke	1695 (12.2)	676 (14.4)	1019 (11.1)
CKD	2485 (17.9)	734 (15.6)	1751 (19.1)
COPD	2236 (16.1)	753 (16.1)	1483 (16.2)
History of heart failure	1188 (8.6)	340 (7.3)	848 (9.2)
Hypertension	10,080 (72.7)	3418 (72.9)	6662 (72.6)
Prior bleed	2456 (17.7)	794 (16.9)	1662 (18.1)
CAD	4151 (29.9)	1302 (27.8)	2849 (31.0)
*In patients with CAD*:			
Angina	2553 (61.5)	828 (63.6)	1725 (60.6)
Myocardial infarction	1867 (45.0)	631 (48.5)	1236 (43.4)

CAD, coronary artery disease; CKD, chronic kidney disease; COPD, chronic obstructive pulmonary disease; LER, lower-extremity revascularization; PAD, peripheral artery disease.

### Incidence of PAD-related revascularization and complications

The overall incidence rate of first PAD-LER was 41.2 per 1000 PYRs (95% CI: 40.5–41.9), and higher in men (45.0 per 1000 PYRs; 95% CI: 44.1–45.9) than women (35.5 per 1000 PYRs; 95% CI: 34.5–36.5). The incidence rate was nearly double for the first endovascular PAD-LER (27.3 per 1000 PYRs; 95% CI: 26.7–27.8) compared to that of open surgery (13.9 per 1000 PYRs; 95% CI: 13.6–14.3).

Among PAD-related complications, all-cause hospitalizations (715.7 per 1000 PYRs; 95% CI: 696.3–735.6) were over three times more common than CV hospitalizations (205.7 per 1000 PYRs; 95% CI: 198.8–212.8) and seven times the rate of revascularization reoccurrence (101.1 per 1000 PYRs; 95% CI: 98.2–104.1). Also, CV mortality accounted for one-third (34.1 per 1000 PYRs; 95% CI: 32.7–35.5) of all-cause mortality (87.0 per 1000 PYRs; 95% CI: 84.8–89.4) (Table 2). The median (IQR) time from first PAD-LER to death was 3.3 years (IQR 5 years).

**Table 2.** Annual incidence rates of peripheral artery disease-related complications.

**Complication**	per 1000 person-years (95% CI)
Acute limb ischemia	26.4 (25.1–27.8)
All-cause hospitalization	715.7 (696.3–735.6)
All-cause mortality	87.0 (84.8–89.4)
Amputation	23.7 (22.5–25.0)
Bleeding	41.0 (39.5–42.7)
Cardiovascular death	34.1 (32.7–35.5)
Cardiovascular hospitalization	205.7 (198.8–212.8)
Myocardial infarction	30.3 (28.9–31.7)
Revascularization reoccurrence	101.1 (98.2–104.1)
Stroke	28.3 (27.0–29.7)
Venous thromboembolism	8.7 (8.0–9.5)

### Treatment patterns

The overall use of all classes of medications increased on or after revascularization with a higher use of nonsteroidal antiinflammatory drugs (NSAIDs), including aspirin (Table 3). A sensitivity analysis of NSAIDs excluding aspirin showed a reduction in use of approximately 40% during the year before the index date and 30% at or after the index date. In the a priori defined subgroups of interest, the proportion on treatment was higher (*p* < 0.05) in older patients and in those with a history of DM, CAD or CKD, with an increase in the proportion on treatment after revascularization. However, prior to revascularization occurrence, NSAID use was similar in patients with or without a history of DM and higher (*p* < 0.05) in patients without a history of CKD.

**Table 3.** Treatment patterns among patients with peripheral artery disease-related lower-extremity revascularization.

Medication use, *n* (%)	All patients (*n* = 13,869)	
	During the year prior to the index date	On or after the index date
Diuretics	3106 (22.4)	3555 (25.6)
Nonsteroidal antiinflammatory drugs^a^	9267 (66.8)	9934 (71.6)
Anticoagulants	1225 (8.8)	2684 (19.4)
ACE inhibitors	5807 (41.9)	6665 (48.1)
Antithrombotics/antiplatelets^a^	9922 (71.5)	11,658 (84.1)
Angiotensin II receptor blockers	1856 (13.4)	2307 (16.6)
Angiotensin receptor neprilysin inhibitors	N/A	21 (0.2)
Beta-blockers	4061 (29.3)	4864 (35.1)
Calcium channel blockers	5285 (38.1)	6310 (45.5)
Lipid-lowering agents	10,540 (76.0)	11,654 (84.0)

a Aspirin included in two categories with differing indications for use.

ACE, angiotensin-converting enzyme; N/A, not applicable.

### Health resource utilization associated with PAD-related complications

Over 93% of patients with PAD-LER were hospitalized for any reason and the commonest reasons for hospitalization were CV diseases (includes coronary heart disease, angina, heart attack, and hypertension) and PAD (includes some codes used to define acute limb ischemia or revascularlization reoccurrence) with a quite high proportion of patients (*n* = 4618) hospitalized for revascularization reoccurrence. Most patients had one to four hospitalization events; the longest mean length of stay was for amputation, followed by stroke and then revascularization reoccurrence (Table 4). Looking at subgroups of interest, patients who underwent a first open surgery revascularization were more often hospitalized for revascularization reoccurrence, amputation, and stroke; those who underwent an endovascular procedure were more often hospitalized for CV events, PAD, and ALI (online Supplementary Table 1). Open surgery procedures were associated with longer length of stay for almost all PAD complications. In particular, the mean length of stay for revascularization reoccurrence was almost double that of the patient group with endovascular revascularization (online Supplementary Tables 3 and 4). Among patients with a history of DM, the commonest reasons for hospitalization were revascularization reoccurrence, amputation, MI, and stroke (online Supplementary Table 1).

**Table 4.** Primary reasons of hospitalization related to PAD complications.

Primary diagnosis	Number (%) of hospitalized patients				Length of stay (days)	
	Total hospitalizations	1–4 hospitalizations	5–9 hospitalizations	10+ hospitalizations	Median (IQR)	Mean (SD)
All-cause	13,014 (93.8)	6677 (51.3)	3562 (27.4)	2775 (21.3)	12 (46)	43.0 (80.4)
CV	11,067 (79.8)	9861 (89.1)	1040 (9.4)	166 (1.5)	4 (15)	15.7 (32.4)
PAD	7318 (52.8)	7114 (97.2)	193 (2.6)	11 (0.2)	2 (8)	10.4 (22.8)
Revascularization reoccurrence	4618 (33.3)	4243 (91.9)	358 (7.8)	17 (0.3)	7 (22)	21.4 (41.6)
Acute limb ischemia	3345 (24.1)	3297 (98.6)	46 (1.4)	< 5	2 (7)	8.5 (18.6)
Amputation	1582 (11.4)	1547 (97.8)	35 (2.2)	−	26 (44)	43.9 (59.0)
Myocardial infarction	866 (6.2)	861 (99.4)	< 10	−	8 (12)	12.3 (14.9)
Stroke	762 (5.5)	762 (100)	−	−	13 (35)	26.8 (33.3)
Bleeding	706 (5.1)	704 (99.7)	< 5	−	3 (10)	9.9 (18.3)
Venous thromboembolism	119 (0.9)	119 (100)	−	−	6 (10)	11.8 (19.0)

CV, cardiovascular (includes coronary heart disease, angina, heart attack, and hypertension); PAD, peripheral artery disease (includes some codes used to define acute limb ischemia or revascularlization reoccurrence).

Patients with CAD history were hospitalized mainly for CV diseases, MI, and stroke (online Supplementary Table 2) whereas those with history of CKD had more hospital admissions for amputation, MI, and bleeding (online Supplementary Table 2). The highest proportion of PAD-LER patients visited general practice for PAD (Table 5) and a similar pattern was confirmed from the subgroup analyses (online Supplementary Tables 5 and 6). Patients with history of DM had more GP visits for ALI, amputation, and MI (online Supplementary Table 5) whereas those with CKD history had more consultations for amputation (online Supplementary Table 6). Compared to patients with a first endovascular revascularization, those who underwent an open surgery procedure visited general practice more frequently for ALI, stroke, amputation, and VTE (online Supplementary Table 5). Current smokers had more GP consultations for PAD, ALI, and revascularization reoccurrence (online Supplementary Table 6) whereas patients with CAD history confirmed the same pattern observed for hospitalization with a greater number of GP visits for stroke, MI, and CV diseases (online Supplementary Table 6).

**Table 5.** General practitioner visits related to PAD complications.

Reason	Number (%) of patient visits			
	Total visits	1–4 visits	5–9 visits	10+ visits
CV	837 (6.0)	752 (89.9)	79 (9.4)	6 (0.7)
PAD	8008 (57.7)	5736 (71.6)	1282 (16.0)	990 (12.4)
Revascularization reoccurrence	1557 (11.2)	1463 (94.0)	62 (4.0)	32 (2.0)
Acute limb ischemia	1740 (12.6)	1480 (85.1)	141 (8.1)	119 (6.8)
Amputation	1471 (10.6)	1214 (82.5)	163 (11.1)	94 (6.4)
Myocardial infarction	884 (6.4)	767 (86.8)	63 (7.1)	54 (6.1)
Stroke	1397 (10.1)	1109 (79.4)	196 (14.0)	92 (6.6)
Bleeding	1301 (9.4)	1234 (94.9)	52 (4.0)	15 (1.1)
Venous thromboembolism	549 (4.0)	502 (91.4)	35 (6.4)	12 (2.2)

CV, cardiovascular (includes coronary heart disease, angina, heart attack, and hypertension); PAD, peripheral artery disease (includes some codes used to define acute limb ischemia or revascularlization reoccurrence).

More GP visits for ALI, stroke, amputation, MI, and VTE were observed in the group of patients undergoing open surgery revascularization. In contrast, patients undergoing endovascular revascularization showed more GP visits for PAD, revascularization reoccurrence, and CV event or follow-up (online Supplementary Tables 7 and 8).

Most patients visited vascular surgery followed by general surgery in the outpatient setting. Also, about 60% of the cohort visited accident and emergency (A&E) for any cause. The actual proportions for each cause are quite small and the most common was cardiac followed by a respiratory condition (Table 6). Similar patterns were confirmed from the subgroup analyses and where some visits were more common than others for a given subgroup analysis, this was as expected given the subgroup investigated.

**Table 6.** Outpatient and accident and emergency visits related to peripheral artery disease complications.

	Number (%) of patient visits			
Treatment specialty	Total visits	1–4 visits	5–9 visits	10+ visits
Outpatient setting*				
Vascular surgery	10,172 (73.3)	5704 (56.1)	2792 (27.4)	1676 (16.5)
General surgery	7007 (50.5)	4656 (66.4)	1624 (23.2)	727 (10.4)
Cardiology	4898 (35.3)	3469 (70.8)	853 (17.4)	576 (11.8)
General medicine	2931 (21.1)	2360 (80.5)	359 (12.3)	212 (7.2)
Urology	2587 (18.7)	1767 (68.3)	515 (19.9)	305 (11.8)
Ear, nose, and throat (ENT)	2196 (15.8)	1757 (80.0)	262 (11.9)	177 (8.1)
Gastroenterology	1985 (14.3)	1714 (86.4)	201 (10.1)	70 (3.5)
Geriatric medicine	1543 (11.1)	1342 (87.0)	137 (8.9)	64 (4.1)
Clinical hematology	1289 (9.3)	775 (60.1)	226 (17.5)	288 (22.4)
Rheumatology	1127 (8.1)	751 (66.6)	195 (17.3)	181 (16.1)
Neurology	1001 (7.2)	860 (85.9)	92 (9.2)	49 (4.9)
Interventional radiology	857 (6.2)	839 (97.9)	16 (1.9)	< 5
Upper gastrointestinal surgery	349 (2.5)	323 (92.6)	22 (6.3)	< 5
Transient ischemic attack	323 (2.3)	321 (99.4)	< 5	−
Orthotics	323 (2.3)	254 (78.6)	54 (16.7)	15 (4.7)
Cardiac surgery	310 (2.2)	295 (95.2)	11 (3.5)	< 5
Cardiothoracic surgery	310 (2.2)	283 (91.3)	24 (7.7)	< 5
Accident & emergency	303 (2.2)	289 (95.4)	10 (3.3)	< 5
Stroke medicine	191 (1.4)	184 (96.3)	< 10	< 5
Cardiac rehabilitation	72 (0.5)	44 (61.1)	15 (20.8)	13 (18.1)
Prosthetics	38 (0.3)	18 (47.4)	< 10	14 (36.8)
Radiology	12 (0.1)	12 (100)	−	−
***Accident and emergency***				
All causes	8303 (59.9)	6056 (72.9)	1557 (18.8)	690 (8.3)
Cardiac conditions	1614 (11.6)	1557 (96.5)	45 (2.8)	12 (0.7)
Respiratory conditions (nonasthma)	1228 (8.9)	1171 (95.4)	48 (3.9)	< 10
Urological conditions	871 (6.3)	847 (97.2)	21 (2.4)	< 5
Vascular injury/other vascular conditions	732 (5.3)	729 (99.6)	< 5	−
Cerebrovascular conditions	537 (3.9)	534 (99.4)	< 5	−
Myocardial ischemia/infarction	464 (3.4)	452 (97.4)	< 10	< 5
Central nervous system conditions	319 (2.3)	317 (99.4)	< 5	−
ENT conditions	249 (1.8)	248 (99.6)	< 5	−
Gastrointestinal hemorrhage	182 (1.3)	182 (100)	−	−
Amputation	< 10	< 10	−	−

* Outpatient codes were defined according to types of clinics as defined in the Clinical Practice Research Datalink (CPRD) GOLD and Aurum primary care databases. Dash (-) indicates none.

Comparing the patients by type of procedure, a significantly higher percentage of patients undergoing open surgery visited general surgery in the outpatient setting whereas a higher percentage of endovascular patients visited interventional radiology. Also, a higher percentage of open surgery patients visited A&E for vascular injury/other vascular conditions compared to endovascular patients (online Supplementary Tables 9 and 10).

### Risk of PAD-related complications among subgroups of interest

Patients with a first open surgery revascularization had a higher risk of most complications (i.e., stroke, amputation, ALI, VTE, CV death, all-cause death), and a lower risk of revascularization reoccurrence. Also, patients with an history of DM or CAD had a higher risk of MI, CV hospitalization, CV death. History of DM was also associated with a higher risk of amputation, ALI, all-cause hospitalization, and all-cause death. A higher risk of stroke was observed in patients older than 50 years. Compared to current smokers, former smokers had a lower risk of ALI and all-cause death, as well as a higher risk of bleeding. Also, patients older than 50 years or with CKD history had a higher risk of CV death and all-cause death (Table 7).

**Table 7.** Risk of peripheral artery disease-related complications among subgroups of interest.

	**Adjusted hazard ratio (95% CI)**
***Risk of stroke***	
Age > 50 years vs age ⩽ 50 years	1.4 (1.1–1.9)
Open surgery vs endovascular revascularization	1.3 (1.1–1.4)
***Risk of amputation***	
Open surgery vs endovascular revascularization	1.4 (1.3–1.6)
History vs no history of diabetes	3.1 (2.8–3.5)
***Risk of acute limb ischemia***	
Former vs current smokers	0.7 (0.6–0.8)
Open surgery vs endovascular revascularization	1.8 (1.6–2.0)
History vs no history of diabetes	1.5 (1.4–1.7)
***Risk of venous thromboembolism***	
Open surgery vs endovascular revascularization	1.2 (1.0–1.5)
***Risk of revascularization reoccurrence***	
Open surgery vs endovascular revascularization	0.8 (0.7–0.9)
***Risk of myocardial infarction***	
History vs no history of diabetes	1.5 (1.3–1.7)
History vs no history of CAD	2.6 (2.3–3.1)
***Risk of bleeding***	
Former vs current smokers	1.3 (1.1–1.5)
***Risk of cardiovascular death***	
Age > 50 years vs age ⩽ 50 years	2.3 (1.7–3.2)
Open surgery vs endovascular revascularization	1.3 (1.2–1.4)
History vs no history of diabetes	1.5 (1.4–1.7)
History vs no history of CAD	1.2 (1.1–1.3)
History vs no history of CKD	1.2 (1.1–1.3)
***Risk of all-cause death***	
Age > 50 years vs age ⩽ 50 years	3.1 (2.5–3.8)
Former vs current smokers	0.8 (0.8–0.9)
Open surgery vs endovascular revascularization	1.1 (1.1–1.2)
History vs no history of diabetes	1.5 (1.4–1.6)
History vs no history of CKD	1.2 (1.1–1.2)
***Risk of cardiovascular hospitalization***	
History vs no history of diabetes	1.2 (1.1–1.3)
History vs no history of CAD	1.2 (1.1–1.3)
***Risk of all-cause hospitalization***	
History vs no history of diabetes	1.3 (1.2–1.4)

CAD, coronary artery disease; CKD, chronic kidney disease.

## Discussion

The results of this large observational study in patients with PAD showed a high annual incidence of lower-limb revascularization which was associated with an increased risk of all-cause hospitalization and mortality. We found the incidence of PAD-LER was higher in men, and in line with clinical evidence^9^ associated with history of one or more conventional risk factors of PAD (i.e., hyperlipidemia, hypertension, DM and CAD). We found an increasing trend in the proportion of PAD-LER as deprivation increased, in keeping with previous PAD^10^ studies, and suggesting that individuals with lower-socioeconomic status remain at high risk – highlighting the need for education and advocacy efforts focused on this at-risk population.^11,12^

Generally, revascularization (either endovascular or surgical) is the treatment of choice in symptomatic patients^13^ but with the advent of improved technology and widespread accessibility, endovascular therapy is fast becoming the first-line treatment. Although surgical patients are generally sicker and have more severe disease,^14^ clinical failures in endovascular therapy also remain high because high-risk patients are sometimes offered endovascular treatment. Accordingly, our findings reported a nearly double incidence of endovascular first revascularization compared with open surgery, a high rate of revascularization recurrence, and a high risk of complications. In our study, patients who had undergone open surgery revascularization had an increased risk for almost all PAD-related complications, suggesting that this type of procedure may be common among patients with more severe disease or be less effective than endovascular therapy. However, it is noteworthy that surgical revascularization was associated with higher risk of amputation compared to an endovascular approach, in keeping with current literature.^15,16^

Not surprisingly, we found that the proportion of patients prescribed each treatment increased on or after the revascularization and we observed a high use of medications routinely used to reduce the incidence of acute events related to thrombosis (i.e., antithrombotic and antiplatelet therapy). As expected, NSAID use was also high and more than 70% of patients were treated with lipid-lowering agents.

The VOYAGER PAD trial,^17^ which involved a broad population of patients who had undergone lower-extremity revascularization, reported that nearly one in five patients in the placebo group had the primary composite outcome of ALI, major amputation for vascular causes, MI, ischemic stroke, or death from CV causes at 3 years. Our results confirm that patients with symptomatic PAD who have undergone lower-extremity revascularization are at high risk for MACE and MALE. A very recent systematic review of 16 randomized controlled trials (RCTs)^18^ highlighted that despite currently available antithrombotic treatments, patients with PAD following revascularization are still at risk for MACE and MALE, stating the need for high-quality studies, better treatment recommendations, and new treatment options that will help guide treatment and optimize care for patients with symptomatic PAD undergoing revascularization.

Our findings highlighted that having a history of DM, CAD, or CKD was associated with higher risk for most of the common PAD-related complications, including adverse CV events. It is known that several comorbidities often coexist in patients with PAD,^19^ leading to a more rapid disease progress, adverse outcomes, and a poor prognosis.^20,21^

The high utilization of healthcare resources observed in our study was more commonly related to PAD and/or CV diseases and consistent with the more severe disease profile of patients with PAD, treated with peripheral revascularization. One-third of patients were hospitalized for revascularization reoccurrence and the longest length of stay was observed for amputation, stroke, and revascularization reoccurrence. Our results suggest that the high-risk subset of patients with PAD, with lower-extremity revascularization places a high economic burden on healthcare systems.

Our study complements information obtained from the National Vascular Registry (NVR).^22^ Although direct comparison is difficult, as the NVR is a registry and differs from our study which captures routinely recorded electronic healthcare record data, we found similar distributions with respect to age and sex for patients undergoing endovascular lower-limb revascularization. In both studies, these patients were reported to have a high prevalence of hypertension and DM and were prescribed antihypertensives, antiplatelets, and statins. We observed a higher percentage of current smokers than the NVR, which instead reported a higher percentage of former smokers. This can be explained by the fact that former smoking is underestimated in CPRD due to a lower degree of completeness of the GP self-reported data.

Assuming that care reported in NVR is likely to be better than that recorded in routine data and that our study may not necessarily capture over-the-counter medications justifying different proportions, we observed that ~72% and 76% of patients were prescribed treatment during the year prior to the first revascularization, with antithrombotics/antiplatelets (including aspirin) and lipid-lowering agents, respectively. These percentages increased to 84% for both medications on or after the first revascularization, in keeping with what is seen in the NVR.

Our results pose the need for more investigation into the mechanisms by which comorbidities influence disease severity and for the development of tailored treatments acting on these pathways. Intervention on lifestyle and targeted therapy of known risk factors could be the key to managing the disease and avoid the massive additional socioeconomic burden deriving from a worsening in its severity.

## Study strengths and limitations

One of the strengths of this manuscript is the data beyond 30 days in addition to socioeconomic status (SES) data, neither of which are included in the NVR.

The main strength of this study is the use of routine clinical data to provide evidence of the burden of PAD-LER in England, confirming results from previous RCTs and providing useful insights for clinical practice. Albeit the undeniable advantages of using electronic healthcare databases such as CPRD and HES, there are some inherent limitations. A major limitation includes the potential for misclassification of diseases and of the outcomes as symptom status, presentation status, and anatomy/severity are not known. Many of the definitions and algorithms to identify patients with the conditions and the complications that are proposed in this study make use of both the CPRD primary care and HES data to increase completeness. Wherever possible, definitions and algorithms that have been validated in these data sources are preferentially used to identify both the diseases of interest as well as the complications.^23–25^

However, due to the low specificity/granularity of the coding, it was not possible to describe patients based on some specific characteristics (i.e., elective vs nonelective patients, major vs minor amputation).

Complications related to PAD were selected based on evidence from the literature and assumed to be related to PAD if they occurred on or after PAD diagnosis. However, this may overestimate the contribution of PAD to complications as some of these events may not be related to PAD. CPRD captures medications that are prescribed to patients. Prescriptions are one step removed from dispensations and the fact that the patient received a prescription for a medication does not ensure that the prescription is actually filled or the patient even took the medication. In addition, over-the-counter medication use or medications administered during hospitalizations is not captured.

## Conclusion

In patients with PAD, the annual incidence of lower-limb revascularization is high and associated with history of one or more conventional PAD-related risk factors. This study highlights a high utilization of healthcare resources and an increased demand for therapy among patients with PAD-LER. Overall, these results suggest a progressive worsening of the life expectancy of these patients, which imposes a high socioeconomic burden.

## Supplemental Material

sj-pdf-1-vmj-10.1177_1358863X221096704 – Supplemental material for Assessment of the burden of disease for patients with peripheral artery disease undergoing revascularization in England Click here for additional data file. Supplemental material, sj-pdf-1-vmj-10.1177_1358863X221096704 for Assessment of the burden of disease for patients with peripheral artery disease undergoing revascularization in England by Laura Portas, Rupert Bauersachs, Kevin Bowrin, Jean-Baptiste Briere, Alexander Cohen, Maria Huelsebeck, Schuyler Jones and Jennifer K Quint in Vascular Medicine
