# Supplementary material for: Assessment of the burden of disease for patients with peripheral artery disease undergoing revascularization in England
Source: Vasc Med. 2022 Jun 7;27(5):440–9. doi: 10.1177/1358863X221096704 (PMC9551319; doi:10.1177/1358863X221096704)
Supplement: sj-pdf-1-vmj-10.1177_1358863X221096704 – Supplemental material for Assessment of the burden of disease for patients with peripheral artery disease undergoing revascularization in England [file sj-pdf-1-vmj-10.1177_1358863X221096704.pdf]

Supplementary Methods

Data Source

The Clinical Practice Research Datalink (CPRD) consists of data collected during consultations in primary care in the UK. With primary care or general practitioners being the first point of contact for medical care in the UK, over 98% of the UK population is registered with a GP[1] and most new symptoms or signs are first picked up in primary care. As of September 2018, only 2.7% of the English primary care registered population had opted out of sharing their confidential patient information for research purposes.[2] Both these factors contribute to CPRD data reaching a broad representativeness of the UK population in terms of age, sex, geographical spread and indices of social deprivation, although with respect to social deprivation, the CPRD population has been shown to be slightly less socioeconomically deprived than the wider population in England (mean decile of 2015 IMD 5.3 in CPRD v. 5.5 in the population of England). 11 CPRD data is also broadly representative of ethnicity and BMI within the UK.[3,4]

At present, CPRD has data for over 13 million current or active patients, which equates to coverage for approximately 20% of the UK population and 15% of UK general practices.[5]

Linked mortality data (date and cause of death) from the Office for National Statistics (ONS), socioeconomic data from the Index of Multiple Deprivation (IMD), and secondary care data from Hospital Episode Statistics (HES) were provided for this study by CPRD for patients in England.

Statistical Analyses

In the calculation of the incidence rate of PAD-related revascularizations over the study period, the numerator consisted of patients who were newly diagnosed with PAD-related low extremity revascularization (PAD-LER) and the denominator included the person-time (in years) when the patient was at-risk of developing the condition (over the time period of interest). In order to ensure that patients with pre-existing revascularization were not included, patients who had undergone a procedure used to treat the disease during the baseline period (in CPRD or HES) were removed from the denominator. Person-time started accruing either on the observation period start date or once the patient had met all the eligibility criteria, whichever came last. Person-time accrued until the incident diagnosis, the patient disenrolled from the practice or the

practice disenrolled from CPRD, the patient died, or the end of the study period. Incidence rates were reported per 1,000 person-years (PYRs) with accompanying 95% confidence intervals.

To estimate the incidence rate and the relative risk of individual PAD complications over the study period, the follow-up time started on the index date (i.e. date of first ever revascularization) and continued until the date of the outcome (i.e. first event for each outcome of interest), death (when it is not the outcome), date of disenrollment in the practice or of the practice in CPRD, or the end of the study. Incidence rates were calculated per 1,000 person-years and 95% confidence intervals were reported.

Ethics and disclosure statement

The data is provided by patients and collected by the National Health Service (NHS) as part of their care and support. The interpretation and conclusions contained in this study are those of the authors alone.
A protocol for this research were approved by the Independent Scientific Advisory Committee (ISAC) for MHRA Database Research (protocol number: 20_008R) and the approved protocol was made available to the journal in which this research is published and to the reviewers during peer review. Generic ethical approval for observational research using the CPRD with approval from ISAC has been granted by a Health Research Authority (HRA) Research Ethics Committee (East Midlands – Derby, REC reference number 05/MRE04/87).

Supplemental Table 1. Hospitalizations by age group, history of diabetes, and type of revascularization procedure.

	**Number (%) of hospitalized patients**					
**Primary diagnosis**	**<=50 years (n=657)**	**>50 years (n=13,212)**	**Diabetes (n=4,175)**	**Non diabetes (n=9,694)**	**Open surgery (n=4,691)**	**Endovascular (n=9,178)**
All-cause	616 (93.8)	12,398 (93.8)	3,907 (93.6)	9,107 (93.9)	**4,261 (90.8)**	**8,753 (95.4)**
CV	**549 (83.6)**	**10,518 (79.6)**	**3,262 (78.1)**	**7,805 (80.5)**	**3,385 (72.2)**	**7,682 (83.7)**
PAD	**408 (62.1)**	**6,910 (52.3)**	**2,096 (50.2)**	**5,222 (53.9)**	**2,282 (48.7)**	**5,036 (54.9)**
Revascularization re-occurrence	**258 (39.3)**	**4,360 (33.0)**	**1,491 (35.7)**	**3,127 (32.3)**	**1,735 (37.0)**	**2,883 (31.4)**
Acute limb ischemia	174 (26.5)	3,171 (24.0)	1,040 (24.9)	2,305 (23.8)	**1,040 (22.2)**	**2,305 (25.1)**
Amputation	90 (13.7)	1,492 (11.3)	**819 (19.6)**	**763 (7.9)**	**657 (14.0)**	**925 (10.1)**
Myocardial infarction	48 (7.3)	818 (6.2)	**360 (8.6)**	**506 (5.2)**	304 (6.5)	562 (6.1)
Stroke	33 (5.0)	729 (5.5)	**263 (6.3)**	**499 (5.2)**	**285 (6.1)**	**477 (5.2)**
Bleeding	39 (5.9)	667 (5.1)	206 (4.9)	500 (5.2)	257 (5.5)	449 (4.9)
Venous thromboembolism	<10	114 (0.9)	29 (0.7)	90 (0.9)	41 (0.9)	78 (0.9)

In bold are statistically significant differences among subgroups (P<0.05).

Abbreviations:

CV: cardiovascular; PAD: peripheral artery disease.

Supplemental Table 2. Hospitalizations by history of CAD, smoking status, and history of CKD.

	Number (%) of hospitalized patients					
**Primary diagnosis**	**CAD (n=4,151)**	**Non-CAD (n=9,718)**	**Current smokers (n=5,197)**	**Former smokers (n=4,336)**	**CKD (n=2,485)**	**Non-CKD (n=11,384)**
All-cause	3,898 (93.9)	9,116 (93.8)	**4,878 (93.9)**	**4,119 (95.0)**	**2,302 (92.6)**	**10,712 (94.1)**
CV	**3,360 (80.9)**	**7,707 (79.3)**	**4,193 (80.7)**	**3,589 (82.8)**	**1,895 (76.3)**	**9,172 (80.6)**
PAD	**2,106 (50.7)**	**5,212 (53.6)**	2,881 (55.4)	2,325 (53.6)	**1,150 (46.3)**	**6,168 (54.2)**
Revascularization re-occurrence	1,378 (33.2)	3,240 (33.3)	1,781 (34.3)	1,452 (33.5)	**728 (29.3)**	**3,890 (34.2)**
Acute limb ischemia	991 (23.9)	2,354 (24.2)	1,277 (24.6)	1,120 (25.8)	566 (22.8)	2,779 (24.4)
Amputation	465 (11.2)	1,117 (11.5)	556 (10.7)	461 (10.6)	**343 (13.8)**	**1,239 (10.9)**
Myocardial infarction	**407 (9.8)**	**459 (4.7)**	**276 (5.3)**	**317 (7.3)**	**178 (7.2)**	**688 (6.0)**
Stroke	**268 (6.5)**	**494 (5.1)**	256 (4.9)	228 (5.3)	131 (5.3)	631 (5.5)
Bleeding	214 (5.2)	492 (5.1)	249 (4.8)	214 (4.9)	**159 (6.4)**	**547 (4.8)**
Venous thromboembolism	28 (0.7)	91 (0.9)	39 (0.8)	37 (0.9)	21 (0.9)	98 (0.9)

In bold are statistically significant differences among subgroups (P<0.05).

Abbreviations:

CAD: coronary artery disease; CKD: chronic kidney disease; CV: cardiovascular; PAD: peripheral artery disease.

Supplemental Table 3. Primary reasons of hospitalization related to PAD complications in patients undergoing endovascular revascularization (N=9,178).

	Number (%) of hospitalized patients				Length of stay (days)	
**Primary diagnosis**		**1-4 hospitalizations**	**5-9 hospitalizations**	**10+ hospitalizations**	**Median (IQR)**	**Mean (SD)**
All-cause	8,753 (95.4)	4,535 (51.8)	2,344 (26.8)	1,874 (21.4)	10 (42)	40.1 (79.5)
CV	7,682 (83.7)	6,882 (89.6)	694 (9.0)	106 (1.4)	2 (12)	13.2 (30.6)
PAD	5,036 (54.9)	4,924 (97.8)	106 (2.1)	<10	1 (5)	8.0 (20.1)
Revascularization re-occurrence	2,883 (31.4)	2,683 (93.1)	191 (6.6)	<10	4 (15)	16.1 (34.1)
Acute limb ischemia	2,305 (25.1)	2,275 (98.7)	29 (1.2)	<5	1 (4)	6.5 (16.2)
Amputation	925 (10.1)	899 (97.2)	26 (2.8)	–	25 (45)	43.3 (56.3)
Myocardial infarction	562 (6.1)	559 (99.5)	<5	–	8 (12)	12.1 (14.7)
Stroke	477 (5.2)	477 (100)	–	–	14 (36)	27.9 (34.7)
Bleeding	449 (4.9)	448 (99.8)	<5	–	3 (10)	9.8 (16.7)
Venous thromboembolism	78 (0.9)	78 (100)	–	–	7 (11)	11.3 (18.1)

Abbreviations: PAD: peripheral artery disease; CV: cardiovascular.

Supplemental Table 4. Primary reasons of hospitalization related to PAD complications in patients undergoing open-surgery revascularization (N=4,691).

	Number (%) of hospitalized patients				Length of stay (days)	
**Primary diagnosis**		**1-4 hospitalizations**	**5-9 hospitalizations**	**10+ hospitalizations**	**Median (IQR)**	**Mean (SD)**
All-cause	4,261 (90.8)	2,142 (50.3)	1,218 (28.6)	901 (21.1)	18 (53)	48.7 (82.0)
CV	3,385 (72.2)	2,979 (88.0)	346 (10.2)	60 (1.8)	8 (21)	21.4 (35.4)
PAD	2,282 (48.7)	2,190 (96.0)	87 (3.8)	<10	6 (13)	15.8 (27.0)
Revascularization re-occurrence	1,735 (37.0)	1,560 (89.9)	167 (9.6)	<10	13 (31)	30.2 (50.5)
Acute limb ischemia	1,040 (22.2)	1,022 (98.3)	17 (1.6)	<5	5 (10)	13.0 (22.4)
Amputation	657 (14.0)	648 (98.6)	<10	–	26 (45)	44.8 (62.4)
Myocardial infarction	304 (6.5)	302 (99.3)	<5	–	8 (12)	12.6 (15.2)
Stroke	285 (6.1)	285 (100)	–	–	12 (33)	25.0 (30.7)
Bleeding	257 (5.5)	256 (99.6)	<5	–	3 (11)	10.2 (20.7)
Venous thromboembolism	41 (0.9)	41 (100)	–	–	6 (8)	12.8 (20.9)

Abbreviations: PAD: peripheral artery disease; CV: cardiovascular.

Supplemental Table 5. GP visits by age group, history of diabetes, and type of revascularization procedure.

	Number (%) of visited patients					
**Reason**	**<=50 years (n=657)**	**>50 years (n=13,212)**	**Diabetes (n=4,175)**	**Non diabetes (n=9,694)**	**Open surgery (n=4,691)**	**Endovascular (n=9,178)**
PAD	**439 (66.8)**	**7,569 (57.3)**	**2,236 (53.6)**	**5,772 (59.5)**	**2,541 (54.2)**	**5,467 (59.6)**
Acute limb ischemia	87 (13.2)	1,653 (12.5)	**619 (14.8)**	**1,121 (11.6)**	**838 (17.9)**	**902 (9.8)**
Stroke	53 (8.1)	1,344 (10.2)	431 (10.3)	966 (10.0)	**538 (11.5)**	**859 (9.4)**
Bleeding	73 (11.1)	1,228 (9.3)	371 (8.9)	930 (9.6)	441 (9.4)	860 (9.4)
Revascularization re-occurrence	**90 (13.7)**	**1,467 (11.1)**	467 (11.2)	1,090 (11.2)	**465 (9.9)**	**1,092 (11.9)**
Amputation	78 (11.9)	1,393 (10.5)	**796 (19.1)**	**675 (7.0)**	**607 (12.9)**	**864 (9.4)**
Myocardial infarction	41 (6.2)	843 (6.4)	**347 (8.3)**	**537 (5.5)**	317 (6.8)	567 (6.2)
CV	42 (6.4)	795 (6.0)	249 (6.0)	588 (6.1)	270 (5.8)	567 (6.2)
Venous thromboembolism	26 (4.0)	523 (4.0)	**139 (3.3)**	**410 (4.2)**	**209 (4.5)**	**340 (3.7)**

In bold are statistically significant differences among subgroups ($P<0.05$).

Abbreviations:

GP: general practitioner; PAD: peripheral artery disease; CV: cardiovascular.

Supplemental Table 6. GP visits by history of CAD, smoking status, and history of CKD.

	**Number (%) of visited patients**					
**Reason**	**CAD (n=4,151)**	**Non-CAD (n=9,718)**	**Current smokers (n=5,197)**	**Former smokers (n=4,336)**	**CKD (n=2,485)**	**Non-CKD (n=11,384)**
PAD	**2,181 (52.5)**	**5,827 (60.0)**	**3,360 (64.7)**	**2,433 (56.1)**	**1,285 (51.7)**	**6,723 (59.1)**
Acute limb ischemia	**466 (11.2)**	**1,274 (13.1)**	**701 (13.5)**	**467 (10.8)**	331 (13.3)	1,409 (12.4)
Stroke	**459 (11.1)**	**938 (9.7)**	500 (9.6)	461 (10.6)	272 (11.0)	1,125 (9.9)
Bleeding	379 (9.1)	922 (9.5)	**467 (9.0)**	**462 (10.7)**	235 (9.5)	1,066 (9.4)
Revascularization re-occurrence	**391 (9.4)**	**1,166 (12.0)**	**675 (13.0)**	**467 (10.8)**	**237 (9.5)**	**1,320 (11.6)**
Amputation	431 (10.4)	1,040 (10.7)	511 (9.8)	439 (10.1)	**320 (12.9)**	**1,151 (10.1)**
Myocardial infarction	**484 (11.7)**	**400 (4.1)**	**280 (5.4)**	**334 (7.7)**	172 (6.9)	712 (6.3)
CV	**323 (7.8)**	**514 (5.3)**	**242 (4.7)**	**360 (8.3)**	150 (6.0)	687 (6.0)
Venous thromboembolism	149 (3.6)	400 (4.1)	203 (3.9)	170 (3.9)	106 (4.3)	443 (3.9)

In bold are statistically significant differences among subgroups (P<0.05).
Abbreviations:
GP: general practitioner; CAD: coronary artery disease; CKD: chronic kidney disease; PAD: peripheral artery disease; CV: cardiovascular.

Supplemental Table 7. GP visits related to PAD complications in patients undergoing endovascular revascularization (N=9,178).

	**Number (%) of visited patients**			
**Reason**		**1-4 visits**	**5-9 visits**	**10+ visits**
PAD	5,467 (59.6)	3,966 (72.5)	854 (15.7)	647 (11.8)
Acute limb ischemia	902 (9.8)	776 (86.0)	61 (6.8)	65 (7.2)
Stroke	859 (9.4)	688 (80.1)	118 (13.7)	53 (6.2)
Bleeding	860 (9.4)	814 (94.6)	35 (4.1)	11 (1.3)
Revascularization re-occurrence	1,092 (11.9)	1,048 (96.0)	29 (2.6)	15 (1.4)
Amputation	864 (9.4)	717 (83.0)	94 (10.9)	53 (6.1)
Myocardial infarction	567 (6.2)	493 (86.9)	39 (6.9)	35 (6.2)
CV	567 (6.2)	511 (90.1)	52 (9.2)	<5
Venous thromboembolism	340 (3.7)	314 (92.3)	19 (5.6)	<10

Abbreviations: PAD: peripheral artery disease; CV: cardiovascular.

Supplemental Table 8. GP visits related to PAD complications in patients undergoing open-surgery revascularization (N=4,691).

	Number (%) of visited patients			
**Reason**		**1-4 visits**	**5-9 visits**	**10+ visits**
PAD	2,541 (54.2)	1,770 (69.7)	428 (16.8)	343 (13.5)
Acute limb ischemia	838 (17.9)	704 (84.0)	80 (9.6)	54 (6.4)
Stroke	538 (11.5)	421 (78.3)	78 (14.5)	39 (7.2)
Bleeding	441 (9.4)	420 (95.2)	17 (3.8)	<5
Revascularization re-occurrence	465 (9.9)	415 (89.2)	33 (7.1)	17 (3.7)
Amputation	607 (12.9)	497 (81.9)	69 (11.4)	41 (6.7)
Myocardial infarction	317 (6.8)	274 (86.4)	24 (7.6)	19 (6.0)
CV	270 (5.8)	241 (89.3)	27 (10)	<5
Venous thromboembolism	209 (4.5)	188 (89.9)	16 (7.6)	<10

Abbreviations: PAD: peripheral artery disease; CV: cardiovascular.

Supplemental Table 9. Outpatient and A&E visits related to PAD complications in patients undergoing endovascular revascularization (N=9,178).

	Number (%) of visited patients			
**Treatment specialty**		**1-4 visits**	**5-9 visits**	**10+ visits**
Outpatient setting:				
Vascular Surgery	6,722 (73.2)	3,912 (58.2)	1,794 (26.7)	1,016 (15.1)
General Surgery	4,501 (49.0)	3,086 (68.6)	998 (22.2)	417 (9.2)
Cardiology	3,279 (35.7)	2,307 (70.4)	564 (17.2)	408 (12.4)
General Medicine	1,918 (20.9)	1,540 (80.3)	229 (11.9)	149 (7.8)
Urology	1,722 (18.8)	1,174 (68.2)	341 (19.8)	207 (12.0)
Ear, Nose and Throat (Ent)	1,485 (16.2)	1,169 (78.7)	187 (12.6)	129 (8.7)
Gastroenterology	1,348 (14.7)	1,165 (86.4)	130 (9.7)	53 (3.9)
Geriatric Medicine	999 (10.9)	862 (86.3)	101 (10.1)	36 (3.6)
Clinical Haematology	816 (8.9)	484 (59.3)	162 (19.9)	170 (20.8)
Rheumatology	784 (8.5)	520 (66.3)	135 (17.2)	129 (16.5)
Neurology	658 (7.2)	562 (85.4)	61 (9.3)	35 (5.3)
Interventional Radiology	698 (7.6)	682 (97.7)	14 (2.0)	<5
Upper Gastrointestinal Surgery	221 (2.4)	204 (92.3)	16 (7.2)	<5
Transient Ischaemic Attack	180 (2.0)	179 (99.4)	<5	–
Orthotics	229 (2.5)	177 (77.3)	41 (17.9)	11 (4.8)
Cardiac Surgery	190 (2.1)	179 (94.2)	<10	<5
Cardiothoracic Surgery	199 (2.2)	182 (91.5)	16 (8.0)	<5
Accident & Emergency (A&E)	204 (2.2)	195 (95.6)	<10	<5
Stroke Medicine	111 (1.2)	109 (98.2)	<5	–
Cardiac Rehabilitation	48 (0.5)	32 (66.7)	10 (20.8)	<10
Prosthetics	21 (0.2)	10 (47.6)	<5	<10
Radiology	<10	<10	–	–
Accident and emergency:				
All-causes	5,478 (59.7)	3,993 (72.9)	1,020 (18.6)	465 (8.5)
Cardiac conditions	1,088 (11.8)	1,051 (96.6)	29 (2.7)	<10
Respiratory conditions (non-asthma)	809 (8.8)	765 (94.6)	36 (4.4)	<10

Urological conditions	571 (6.2)	556 (97.4)	13 (2.3)	<5
Vascular injury/Other vascular conditions	406 (4.4)	405 (99.7)	<5	–
Cerebro-vascular conditions	330 (3.6)	328 (99.4)	<5	–
Myocardial ischaemia/Infarction	309 (3.4)	301 (97.4)	<10	<5
Central nervous system conditions	214 (2.3)	213 (99.5)	<5	–
ENT conditions	159 (1.7)	159 (100)	–	–
Gastrointestinal haemorrhage	112 (1.2)	112 (100)	–	–
Amputation	<5	<5	–	–

Abbreviations:
A&E: accident and emergency; PAD: peripheral artery disease; ENT: ear, nose and throat.

Supplemental Table 10. Outpatient and A&E visits related to PAD complications in patients undergoing open-surgery revascularization (N=4,691).

		**Number (%) of visited patients**		
**Treatment specialty**		**1-4 visits**	**5-9 visits**	**10+ visits**
Outpatient setting:				
Vascular Surgery	3,450 (73.5)	1,792 (51.9)	998 (28.9)	660 (19.2)
General Surgery	2,506 (53.4)	1,570 (62.6)	626 (25.0)	310 (12.4)
Cardiology	1,619 (34.5)	1,162 (71.8)	289 (17.8)	168 (10.4)
General Medicine	1,013 (21.6)	820 (81.0)	130 (12.8)	63 (6.2)
Urology	865 (18.4)	593 (68.6)	174 (20.1)	98 (11.3)
Ear, Nose and Throat (Ent)	711 (15.2)	588 (82.7)	75 (10.5)	48 (6.8)
Gastroenterology	637 (13.6)	549 (86.2)	71 (11.1)	17 (2.7)
Geriatric Medicine	544 (11.6)	480 (88.2)	36 (6.6)	28 (5.2)
Clinical Haematology	473 (10.1)	291 (61.5)	64 (13.5)	118 (25.0)
Rheumatology	343 (7.3)	231 (67.3)	60 (17.5)	52 (15.2)
Neurology	343 (7.3)	298 (86.9)	31 (9.0)	14 (4.1)
Interventional Radiology	159 (3.4)	157 (98.7)	<5	–
Upper Gastrointestinal Surgery	128 (2.7)	119 (93.0)	<10	<5
Transient Ischaemic Attack	143 (3.1)	142 (99.3)	<5	–
Orthotics	94 (2.0)	77 (81.9)	13 (13.8)	<5
Cardiac Surgery	120 (2.6)	116 (96.7)	<5	<5
Cardiothoracic Surgery	111 (2.4)	101 (91.0)	<10	<5
Accident & Emergency (A&E)	99 (2.1)	94 (95.0)	<5	<5
Stroke Medicine	80 (1.7)	75 (93.8)	<5	<5
Cardiac Rehabilitation	24 (0.5)	12 (50.0)	<10	<10
Prosthetics	17 (0.4)	<10	<5	<10
Radiology	<10	<10	–	–
Accident and emergency:				
All-causes	2,825 (60.2)	2,063 (73.0)	537 (19.0)	225 (8.0)
Cardiac conditions	526 (11.2)	506 (96.2)	16 (3.0)	<5
Respiratory conditions (non-asthma)	419 (8.9)	406 (96.9)	12 (2.9)	<5

Urological conditions	300 (6.4)	291 (97.0)	<10	<5
Vascular injury/Other vascular conditions	326 (6.9)	324 (99.4)	<5	–
Cerebro-vascular conditions	207 (4.4)	206 (99.5)	<5	–
Myocardial ischaemia/Infarction	155 (3.3)	151 (97.4)	<5	–
Central nervous system conditions	105 (2.2)	104 (99.1)	<5	–
ENT conditions	90 (1.9)	89 (98.9)	<5	–
Gastrointestinal haemorrhage	70 (1.5)	70 (100)	–	–
Amputation	<10	<10	–	–

Abbreviations:
A&E: accident and emergency; PAD: peripheral artery disease; ENT: ear, nose and throat.

Supplementary references

1. Herrett E, Gallagher AM, Bhaskaran K, et al. Data Resource Profile: Clinical Practice Research Datalink (CPRD). International Journal of Epidemiology. 2015;44 (3); 827-36. doi: 10.1093/ije/dyv098.

2. Wolf A, Dedman D, Campbell J, et al. Data resource profile: Clinical Practice Research Datalink (CPRD) Aurum. International Journal of Epidemiology. 2019;48(6):1740-1740g. doi: 10.1093/ije/dyz034.

3. Bhaskaran K, Forbes HJ, Douglas I, et al. Representativeness and optimal use of body mass index (BMI) in the UK Clinical Practice Research Datalink (CPRD). BMJ Open. 2013;3(9): e003389. doi: 10.1136/bmjopen-2013-003389.

4. Mathur R, Bhaskaran K, Chaturvedi N, et al. Completeness and usability of ethnicity data in UK-based primary care and hospital databases. Journal of Public Health (Oxford, England). 2014; 36(4):684-92. doi: 10.1093/pubmed/fdt116.

5. Release Note: CPRD Aurum, June 2021. https://www.cprd.com/sites/default/files/2021-06%20CPRD%20Aurum%20Release%20Notes.pdf [last accessed 5th July 2021].
